# An IoT-Based Smart Home Automation System

**DOI:** 10.3390/s21113784

**Published:** 2021-05-30

**Authors:** Cristina Stolojescu-Crisan, Calin Crisan, Bogdan-Petru Butunoi

**Affiliations:** 1 Communication Department, Politehnica University of Timisoara, 300223 Timișoara, Romania; 2 SafeFleet Telematics, 300223 Timisoara, Romania; ccrisan@gmail.com; 3 Computer Science Department, West University of Timisoara, 300223 Timișoara, Romania; bogdan.butunoi94@e-uvt.ro

**Keywords:** Internet of Things, home automation, smart homes, sensors

## Abstract

Home automation has achieved a lot of popularity in recent years, as day-to-day life is getting simpler due to the rapid growth of technology. Almost everything has become digitalized and automatic. In this paper, a system for interconnecting sensors, actuators, and other data sources with the purpose of multiple home automations is proposed. The system is called qToggle and works by leveraging the power of a flexible and powerful Application Programming Interface (API), which represents the foundation of a simple and common communication scheme. The devices used by qToggle are usually sensors or actuators with an upstream network connection implementing the qToggle API. Most devices used by qToggle are based on ESP8266/ESP8285 chips and/or on Raspberry Pi boards. A smartphone application has been developed that allows users to control a series of home appliances and sensors. The qToggle system is user friendly, flexible, and can be further developed by using different devices and add-ons.

## 1. Introduction

**Figure 1:**
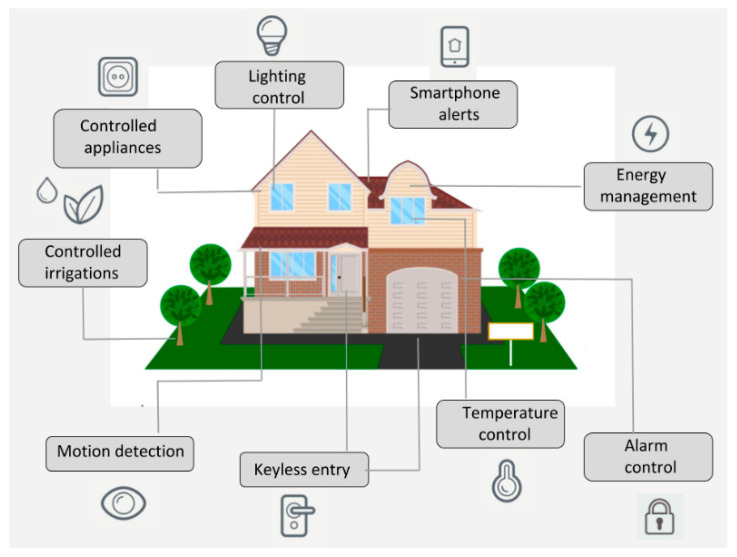
An IoT-based smart home depicting the use of smart sensing devices for different purposes.

The Internet of Things (IoT) is a system that allows devices to be connected and remotely monitored across the Internet. In the last years, the IoT concept has had a strong evolution, being currently used in various domains such as smart homes, telemedicine, industrial environments, etc. [1]. Wireless sensor network technologies integrated into the IoT enable a global interconnection of smart devices with advanced functionalities [2]. A wireless home automation network, composed of sensors and actuators that share resources and are interconnected to each other, is the key technology to making intelligent homes. A “smart home” is a part of the IoT paradigm and aims to integrate home automation. Allowing objects and devices in a home to be connected to the Internet enables users to remotely monitor and control them [3]. These include light switches that can be turned on and off by using a smartphone or by voice command, thermostats that will adjust the indoor temperatures and generate reports about energy usage, or smart irrigation systems that will start at a specific time of a day, on a custom monthly schedule, and thus will control water waste. Smart home solutions have become very popular in the last years. Figure 1 shows an example of a smart home that uses different IoT-connected utilities.

One of the greatest advantages of home automation systems is their easy management and control using different devices, including smartphones, laptops and desktops, tablets, smart watches, or voice assistants. Home automation systems offer a series of benefits; they add safety through appliance and lighting control, secure the home through automated door locks, increase awareness through security cameras, increase convenience through temperature adjustment, save precious time, give control, and save money.

Several home automation systems involved with IoT have been proposed by academic researchers in the literature in the last decade. In wireless-based home automation systems, different technologies have been used, each of them with their pros and cons. For example, Bluetooth-based automation [4,5,6] is low cost, fast, and easy to be installed, but it is limited to short distances. GSM and ZigBee are widely used wireless technologies as well. GSM provides long-range communication at the cost of a mobile plan of the service provider that operates in the area. Zigbee [7,8,9,10,11,12] is a wireless mesh network standard that is designed to be low-cost and with low power consumption, targeted at battery-powered devices in wireless control and monitoring applications. However, it has a low data speed, low transmission, as well as low network stability, and has a high maintenance cost. Wi-Fi technology is used in [9,11,12,13,14,15,16,17,18]. The advantages of Wi-Fi technology over ZigBee or Z-Wave are related to price, complexity (meaning simplicity), and accessibility. First, Wi-Fi-enabled smart devices are usually cheap. In addition, it is easier to find do-it-yourself devices that use Wi-Fi, resulting a less expensive option. Second, Wi-Fi is already a necessity and it is in most homes, so it is easier to buy devices that are already Wi-Fi-enabled. Finally, Wi-Fi is characterized by simplicity, meaning that a user must connect only a minimal number of devices for a home automation setup. Since it is very common, the investment on extra hardware is avoided; a user only needs the basic setup for a home automation system. However, Wi-Fi is not designed to create mesh networks, it consumes ten times more energy than similar devices using ZigBee, Z-Wave, or Bluetooth for example, and many Wi-Fi routers can only allow up to thirty devices connected at once. As compared to Ethernet, Wi-Fi brings several advantages, including the easy connection and access of multiple devices, the expandability (adding new devices without the hassle of additional wiring), lower cost, or single access point requirement. The cons include limited distance to cover (a Wi-Fi network with standard equipment can be limited in range through walls and other obstructions in a standard home), the number of devices can be limited, there is interference and complex propagation effects, obstacles can block the Wi-Fi signal and affect the devices connected to it, and there are connection speed (the fastest speed of Wi-Fi is much slower than a wired network), Internet security, and privacy issues. Low-cost, open source hardware components, such as Arduino and Raspberry Pi microcontroller unit (MCU) boards, and a combination of sensors have been very used in the home automation domain. Home automations using Arduino boards are proposed in [3,19,20,21,22,23]. Arduino is highly flexible, open source, not expensive, and easy to program [19]. In addition, the existence of a large and active community of users is a great plus. However, Arduino is not designed to handle the large complexity that comes with advanced projects. For more advanced and real-time projects, Raspberry Pi is a better option. Raspberry Pi is an exciting technological development that is much cheaper than any desktop computer or mobile device [24]. Most of the software and projects done on Raspberry Pi are open source and are maintained by online user communities, which are always excited about new projects. When developing software on Raspberry Pi, Python is the language of choice, since it is relatively simple (fewer lines and less complexity) compared to other programming languages. In addition to its low price, Raspberry Pi is energy efficient and does not require any cooling systems. Smart home automations with Raspberry Pi are proposed in [9,12,15,25,26]. ESP8266 chips are low-price Wi-Fi modules that are perfectly suited for projects in the IoT field. ESP8266 is a single core processor that runs at 80 MHz. ESP8266 chips were used for home automations-related projects in [9,21,27,28,29,30]. A features comparison for home automation system published in scientific papers, in the last ten years, is presented in Table 1.

Another category of home automation systems is represented by commercial platforms, such as Qivicon, Domintell, Loxone, or HomeSeer. They offer a wide range of smart home devices, from multiple vendors, different communications protocols for wired (Domintell) and wireless (Qivicon) transmissions, or both (HomeSeer and Loxone), and multiple automations such as a locking system, controlling temperature, lightning system, environmental system, video surveillance (only Qivicon and HomeSeer), or anti-intrusion. All solutions provide a mobile app for controlling the systems. Pricewise, it depends on the size of the house, the number of devices to be installed, and the needs of the user. According to [9], the minimum cost comes between 1800 and 2600 euros.

Currently, a great variety of open source home automation systems exist [37,38,39,40,41,42]. OpenHAB [37] and Home Assistant [38] are two of the strongest players in the open source home automation community, sharing a similar vision and integrating many devices. However, openHAB requires knowledge regarding how to insert commands to integrate devices; it is complex and time consuming. Home Assistant, on the other hand, is more user friendly, but it requires a significant configuration effort. Mobile apps seem less flexible and quite complicated and complex, especially for beginners. Domoticz [39] delivers a decent number of features; its configuration is mostly done through the web interface, and plugins are used to extend its functionality. Unfortunately, the interface itself is not extremely intuitive. Domoticz is quite limited in terms of supported devices and configurations. Calaos [40] and Jeedom [41] are two French players in the open source home automation community. Unfortunately, the communities and forums are predominantly French, which can be a barrier to worldwide adoption. A feature comparison of the most relevant open-source home automation platforms is presented in Table 2. The platforms can be differentiated, among others, in terms of the development language, the API, the amount of implemented protocols and plugins, and the amount and type of documentations. Of course, these are not the only options available. The authors of [19] present a detailed comparison of fifteen open-source platforms. 

The purpose of this paper is to present qToggle, which is a system designed and developed for multiple home/building automations, including access control and security, appliances control (lights, thermostats, AC, and other appliances), irrigations, and power and energy management. This paper represents an extension of [43]. In [43], we proposed a building automation solution to reduce the exposure and transmission of COVID-19 during the pandemic situation in workspaces by avoiding touching certain objects and surfaces and for helping managing buildings during an emergency. In this paper, we have focused on smart homes applications, in general, not in a pandemic situation. 

What makes our proposal different from others is highlighted in Table 1: it is different in terms of the technologies used, the controllers, the type of communication, the user interface, and most of all the applications regarding what solutions it can offer in terms of a smart home. The communication technology represents a key point to achieve successful operation in a home automation system. In many papers in the literature, the authors combine several communication technologies; for example, the authors use either a wired or a wireless technology to connect the sensors with the nodes, a wireless technology to send data from nodes to storage centers, etc. Ethernet and/or a Wi-Fi local network are usually enough for a working qToggle setup. Most low-cost devices for IoT usually support Wi-Fi, and most households are able to provide enough wireless coverage with several low-cost devices. The best node and the selection of the processor (controller) for an IoT-based home automation system are chosen considering the necessities and the characteristics a user wants for the system. Even if most automation systems presented in the literature use Arduino boards, the Raspberry family are frequently used as well, since they are more potent than the Arduino boards and have powerful computing abilities that allow the implementation of more demanding software and algorithms. Hence, we chose the Raspberry Pi board for the proposed system. Our system has not only a research scope, but we intended to develop a system that can be successfully used in practice and as well as monetized in the future. The microcontroller used for the proposed system is the ESP8266 chip, due to its size, ultralow power consumption, powerful on-board processing, and storage.

Most systems do not have access to the power grid or may only receive power during a given time period. The use of solar energy reduces the energetic costs, which is an advantage for home automation systems. Starting this year, the qToggle system proposed in this paper will be using solar energy thanks to the photovoltaic panels installed, so the energy consumption will not be an issue.

qToggle is built around a flexible and powerful API (we have defined the API from the ground up), allowing various types of devices to work together. qToggle provides a simple language for the IoT by using the JavaScript Object Notation (JSON) data format, which is defined by RFC 7159. Turning on a light bulb should be as easy as PATCH-ing a URL, while obtaining the temperature from a sensor requires a simple GET request. The idea behind qToggle is to control programmable systems having a Transmission Control Protocol/Internet Protocol (TCP/IP) stack via simple HTTP requests. For example, these systems can be single-board computers or TCP/IP-enabled microcontrollers. qToggle aims to propose a standard that allows managing, provisioning, and communicating to different devices. qToggle does not attempt to reinvent the wheel, but it makes use of the existing and widely used technologies, such as RESTful APIs on the top of HTTP, passing over data encoded as JSON.

Features that make qToggle special are the following:

- A unitary and consistent solution that integrates all required features;
- Device provisioning and management;
- The firmware update over the same unique API used by all devices;
- The use of expressions allows intelligent and complex rules to be implemented between various sensors and actuators inside a network;
- Hierarchical master-slave topology that offers flexibility and scalability;
- User data do not leave the premises of the local network, a cloud connection not being needed (for security and privacy reasons);
- The integrated web app works well on all major platforms (both desktop and mobile): Android, iOS, Windows, Linux, or macOS.

## 2. Materials and Methods

### 2.1. System Architecture and Design

The classic Ethernet and/or a Wi-Fi local network are usually enough for a working qToggle setup. The different hardware used in the system includes Raspberry Pi 3 or 4 boards (any model), ESP 8266 Wi-Fi modules, and smart devices. The Raspberry Pi version used for this project is Raspberry Pi 4, due to the improvements brought, as compared to previous versions. For example, Raspberry Pi 1 and 2 do not have Bluetooth (it is needed for controlling the thermostats). An important feature of the Raspberry Pi is the row of general-purpose input/output (GPIO) pins. A 40-pin GPIO header is found on all current Raspberry Pi boards [44]. The three roles of a Raspberry Pi board in a qToggle setup are the following: the board could act as a qToggle device when it is equipped with peripherals (sensors or relay boards), it could also act as a master hub for other devices, and, finally, it could help install the ESP firmware on some devices, when running Tuya Convert OS (Tuya is a Chinese smart devices platform that offers cloud services for ESP8266/ESP8285-based devices). Tuya Convert OS helps replace this proprietary Tuya firmware with a custom firmware, without disassembling the device. An important fact is that it works only for Tuya-based devices. In fact, Tuya Convert OS is a customized Raspbian OS image that runs Tuya Convert with a friendly user interface. 

The main part of the home automation system based on IoT is the microcontroller. The ESP 8266 Wi-Fi module represents a set of efficient highly integrated wireless Systems on Chip (SoCs), which provides a complete and standalone Wi-Fi network solution. The ESP8266EX version is one of the most integrated Wi-Fi chips in the industry. In addition to its Wi-Fi functionalities, ESP8266EX integrates an enhanced version of L106 Diamond series 32-bit processor from Tensilica (company based in Silicon Valley, in the semiconductor domain), with on-chip SRAM. ESP8266EX has seventeen GPIO pins, which can be assigned to various functions by programming the appropriate registers, two power pins, one ground pin, reset pin, and two clock pins. The devices used by qToggle are usually sensors or actuators with an upstream network connection. Keeping the device firmware updated is probably one of the most essential tasks, and it is often neglected when dealing with a large number of devices. qToggle facilitates this task by allowing updates of the firmware very simply for devices of different types and models. The qToggle API is an intuitive HTTP API that enables remote controlling of basic hardware ports, such as GPIOs or analog-to-digital converters (ADC).

The idea behind qToggle is to control programmable systems having a TCP/IP stack via simple HTTP requests. For example, these systems can be single-board computers or TCP/IP-enabled microcontrollers. API functions are grouped into the following categories:

- Device management—general status and configuration of the device;
- Port management—port information and configuration;
- Port values—reading and writing values from and to ports;
- Notifications—event notifications;
- Reverse API calls—API calls via reverse HTTP requests.

API specifications may seem quite complex, offering a wide range of functionalities and use cases. However, most of them are optional, and only a small set of functions are mandatory for a qToggle implementation. 

**Figure 2:**
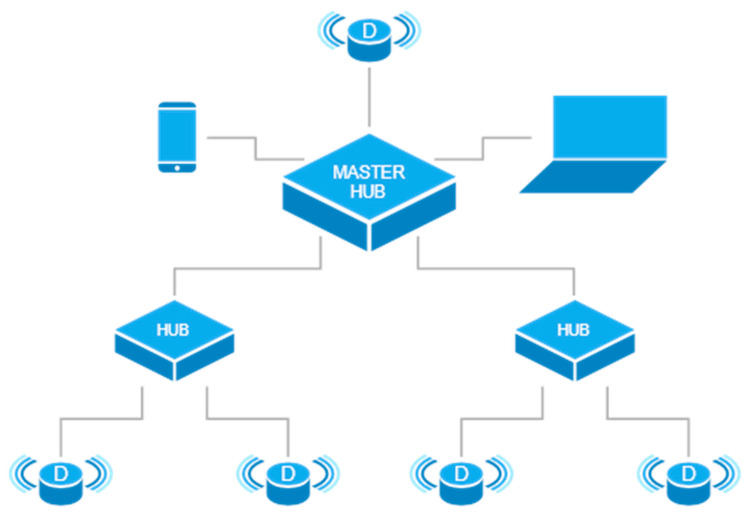
The qToggle topology.

The qToggle ecosystem is composed of a qToggleServer, qToggleOS, espQToggle, add-ons, and other tools and packages that are specific to certain setups and use cases. The main component is qToggleServer, which is written in Python. It acts as a hub and provides the user-friendly web app. qToggleOS is an operating system (OS) ready to be used with Raspberry Pi boards and runs qToggleServer. espQToggle is a custom firmware for ESP8266/ESP8285 devices and implements the qToggle API. Finally, add-ons are optional pieces of software that enhance the functionality of qToggleServer. A device used by qToggle will describe itself, indicating its configuration, its supported optional functionalities, and what ports it exposes. Each port, in its turn, will describe itself, indicating its identifier, type, configuration, and so on. By combining master–slave relationships between simple devices and hubs in a network, a complex tree topology is obtained. Thus, a large number of smart devices can be easily managed, as shown in Figure 2. The type of communication in Figure 2 is Wi-fi or Ethernet. Consumers could operate at any level in the hierarchy, thus limiting the access inside the network to any desired subtree.

In a real-world setup, it is difficult to individually manage many devices. Therefore, a hub will allow a centralized administration of devices used by qToggle. Hubs act as consumers when communicating to other devices, but they also expose an API interface that allows other consumers to see them as devices. This allows for the creation of complex hierarchies of devices and hubs that are in a master–slave relationship. With qToggle, a device may act as a master for other slave devices. The master controls slave devices and allows accessing them through its own API functions. In the same way, a slave device can act as a master for other devices. Thus, complex chained master–slave configurations can be obtained. Special API functions are supported by the master for listing, adding, and removing slaves. Slave devices are identified on the master by their names, but the master must be prepared for a slave’s name to change at any time.

qToggle implements three roles that dictate the access level: the administrator role, which has the absolute power over a device, being able to view and modify the configuration; the normal role, which has no access the configuration but can read from/write to ports, and the view-only role that can only read the port’s values. To facilitate automation, qToggle allows adding rules that dictate port values, based on various conditions. This means that a port can be taught to use an expression based on other ports and functions in a way that resembles spreadsheet formulas. Expressions can be set at a device level or at a hub level. Expressions on a device are very fast, but they can only depend on ports present on the device. When setting an expression at a hub level, the ports of any device that is known by the hub can be included. This will effectively implement relations between different devices. If consumers need to be notified about events that take place on the device, for example port value changes, qToggle offers three notification methods: listening for events using long HTTP requests (long polling), webhooks, and polling (the least efficient, but easiest to be implemented). qToggle setups are usually deployed in private networks, where devices cannot be directly accessed from the Internet. The solutions often depend on port forwarding, where public IPs are available. If port forwarding is not wanted/impossible, the devices can be set to open a connection to an external public server and to wait for API requests. This mechanism is called reverse HTTP and allows making HTTP requests to a device inside a private network without forwarding any port.

From a developer point of view, qToggle offers add-ons that are an easy and convenient way of packaging optional functionalities, which are usually tied to a specific device or service. Add-ons can be published or be kept private, depending on the developer’s needs and licensing requirements. The entire source code is completely opensource [45], so one can easily understand how it works, may propose changes or may even join the team. In addition, we provide documentation on using and further developing qToggle for new devices or use cases.

Regarding the security, qToggle uses a series of best practices that are often found in nowadays web-based applications. HTTPS is employed for when a client from the outside (the Internet) talks to the hub. It ensures encryption, authenticity of the hub, and integrity of the HTTP messages. Plain HTTP is used only locally, inside the premises, between the hub and its controlled devices. A TLS certificate is used in conjunction with HTTPS to ensure the security goals mentioned above; Let’s Encrypt is used to generate and renew the TLS certificates. This process is done automatically on the hub, upon certificate expiry. Remote (administrative) access on the hub is done via SSH. The SSH protocol uses ECDSA (or similar) private/public key pairs for authentication and encryption. Alternatively, the administrator password defined on the hub may be used to log in remotely with username and password.

The API defines three roles that dictate the permissions of an API request: administrator, normal user, and view-only user. API requests use the JSON Web Token (JWT) defined by RFC 7519 to supply authentication data. A shared secret (called password) ensures the authenticity of the caller. The secret is hashed with a salt before used to sign the JWT token to prevent compromising the original password. Reply attacks are prevented by using the current timestamp as a nonce included in the JWT.

Alternatively, we could have used HTTP Basic Authentication, HTTP Digest Authentication, or a cookie-based session management with a conventional login form. Basic Authentication is insecure when transmitted over unencrypted channels, while Digest Authentication is unnecessarily complicated and requires exchanging multiple messages. The cookie/session-based method is prone to session stealing attacks and may also be insecure on unencrypted channels.

The embedded Over-the-Air (OTA) mechanism (firmware update) ensures that the hub as well as its attached devices always run the latest available version, thus allowing us to quickly bring security patches in case a vulnerability is discovered.

### 2.2. Configuring the Web Application

**Figure 3:**
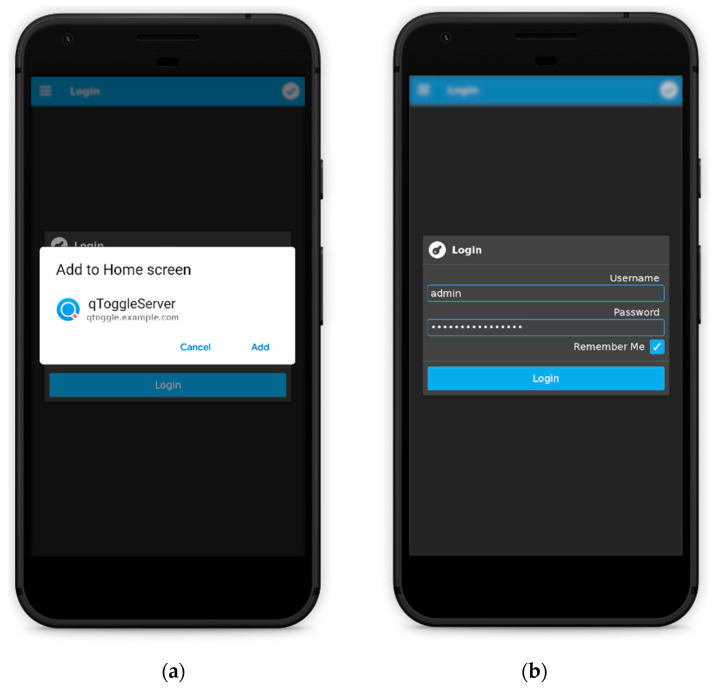
Logging in for the first time on qToggleServer (**a**) and setting used and password (**b**).

qToggleServer provides a user-friendly interface, named frontend, which comes in the form of a progressive web application (PWA). It is designed to be used on smartphones, tablets, but also on laptops/desktop machines. Firstly, the application should be installed and, being a PWA, it should be added to the home screen. After installation, the qToggle app will be found in the applications list of the device, and it can be uninstalled whenever the user wants to. When the user logs in for the first time (see Figure 3a), an admin with an empty password should be used. However, for security reasons, it is highly recommended to set a password in the Settings page of the app.

**Figure 4:**
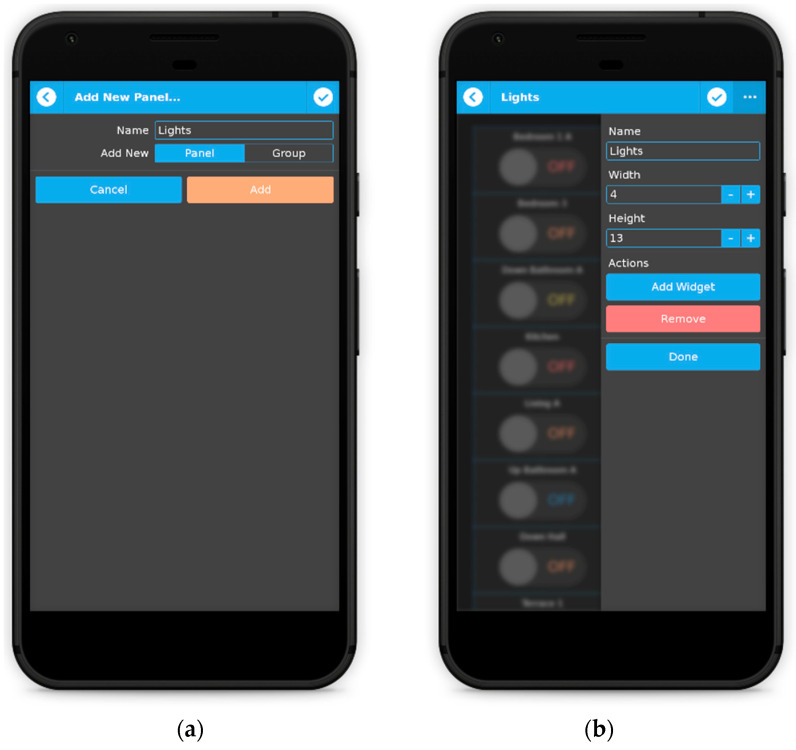
Creating panels (**a**) and groups of panels (**b**).

The dashboard is the section where users will spend most of the time when using qToggleServer. Here, they can create panels and groups of panels, as shown in Figure 4. In the panel edit mode, the user can perform various tasks, for example add, move around, remove, resize, or configure widgets. Widgets usually require selecting one or more ports. Ports values will be displayed and/or changed by the widget upon interaction.

**Figure 5:**
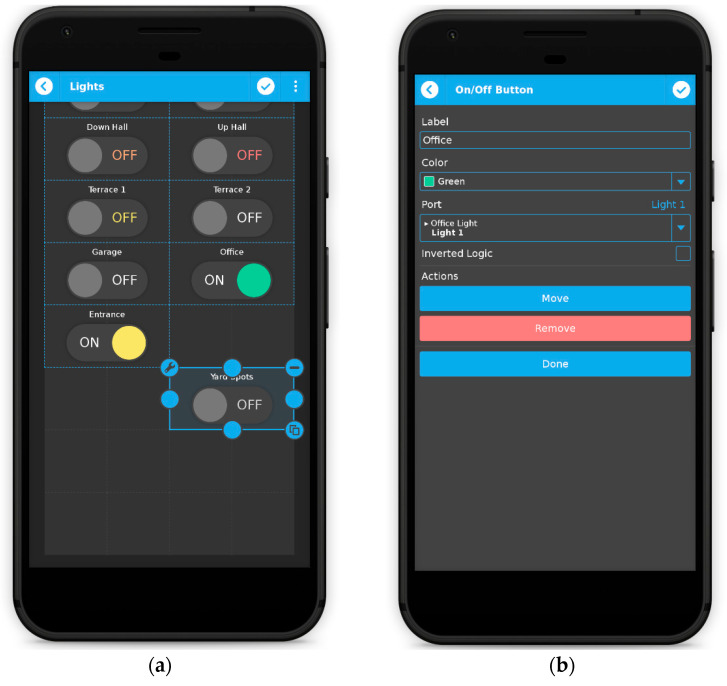
Working with widgets: dashboard layout (**a**), widget properties (**b**).

**Figure 6:**
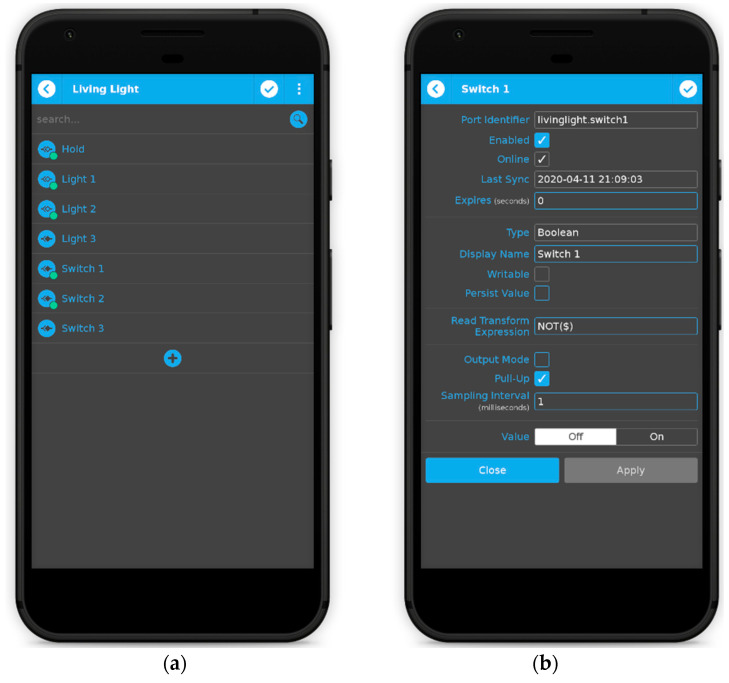
Adding, removing (**a**) and configuring ports (**b**).

**Figure 7:**
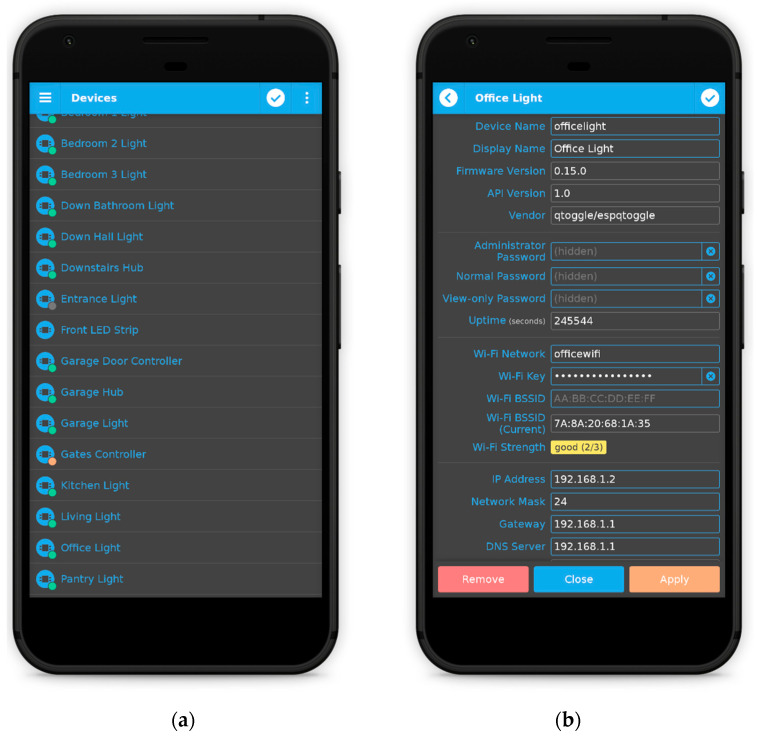
Adding, removing (**a**) and configuring slave devices (**b**).

An example is given in Figure 5. The ports section is only accessible to administrators. In this section, the user may add, remove, and configure ports (see Figure 6). If users have slave devices management enabled in qtoggleserver.conf (by default they are enabled), the first thing they will have to do is to select the device whose ports will be edited. The first device in the list represents the hub (the master device) itself. An important fact is that only administrators can add, remove, and configure slave devices (see Figure 7). 

qToggle app is linked with the qToggleServer package. This means that users will get an app update whenever they update their qToggleServer installation. Since qToggle is a web app, the update process is done automatically by the browser, when the user reopens or refreshes the app. The user can either close it and reopen it, or he/she can use the pull-to-refresh function to make sure the app is up to date. The code and documentation for qToggle can be found on Github [45].

## 3. Real Home Case Study

In the following, the use of qToggle in a real home will be presented. The scenario consists of a two-floor house with five rooms, two bathrooms, kitchen, pantry, shed, garage, and garden. In this case, qToggle is used for various purposes, such as:

- Controlling the indoor temperature (thermostats and air conditioning (A/C));
- Controlling the lights (on–off);
- Monitoring the power and the energy;
- Controlling the doors—gates, garage door, or both at the same time (open–close);
- Security—the alarm;
- Garden sprinklers.

### 3.1. Controlling Temperatures and A/C

**Figure 8:**
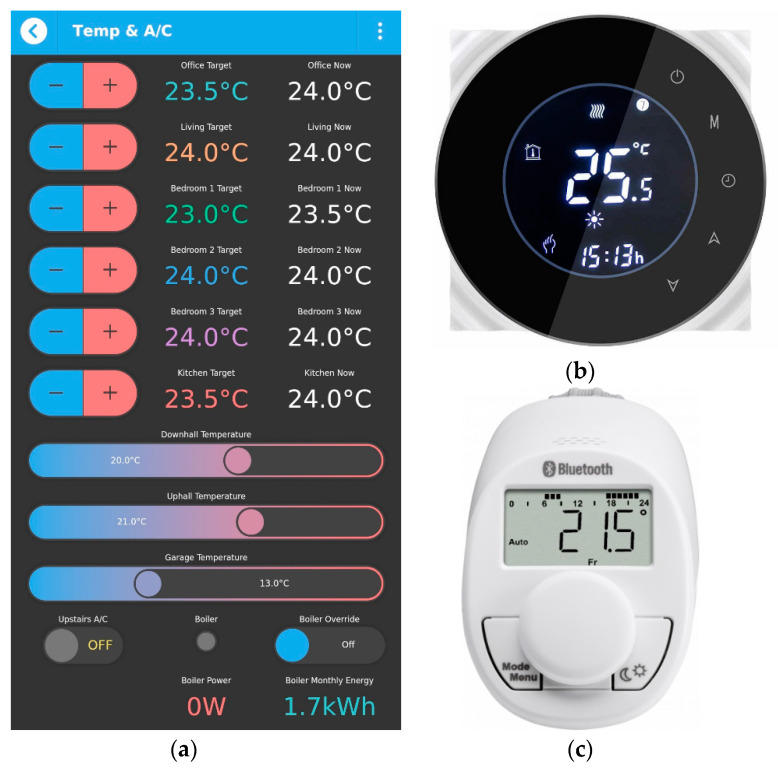
Controlling indoor temperatures with qToggle app (**a**) together with smart termostats (**b**,**c**).

The purpose of controlling the indoor temperatures is to maintain thermal comfort and to save energy cost. In this case, the thermostats system offers the following advantages: the ability to access and control the indoor temperature anytime and from anywhere using qToggle app on the mobile phone, as presented in Figure 8a, the ability to monitor and separately set the temperature in individual rooms (not every room has the same heating requirements), and, finally, the ability to enable scheduling (lower the temperature during the day, when nobody is home, or during vacation). In this way, manual adjustments are eliminated to save time and effort. 

This case study home is provided with nine smart thermostats. Thanks to temperature sensors, the heat system will start only when the temperature falls under a set value (this value is set on qToggle app, for each room). For this project, six Smart Wi-Fi Touch Thermostat Temperature Wireless Controllers, connected to the power line (shown in Figure 8b) and three Eqiva’s eq-3 Bluetooth smart thermostats, which run on batteries, (shown in Figure 8c) have been used. 

The A/C can be controlled to turn on and off without using the remote, by using the qToggle app. This task can be done using a smart plug for the A/C machine. Controlling the degrees is a feature that can be very easily implemented in qToggle, if necessary, and it will look similar to the case of thermostats. 

### 3.2. Controlling the Lights

**Figure 9:**
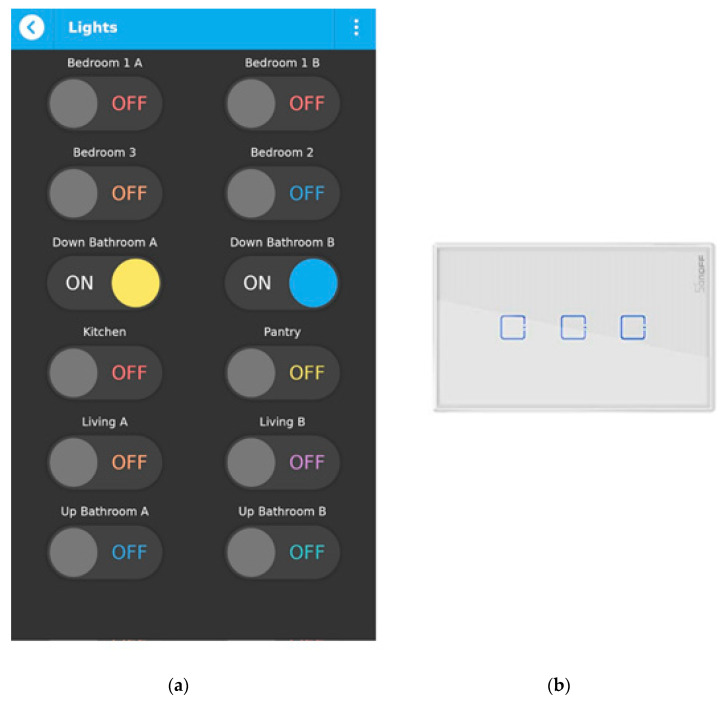
Switching on and off the lights in a house with qToggle app (**a**) and the Sonoff Touch device (**b**).

The proposed lighting control system on the qToggle app is shown in Figure 9. One of its main advantages is, of course, the comfort. Smart lights can, without any doubts, make our everyday life easier. Another advantage is related to energy saving. Big houses, with many rooms, can waste a lot of energy by simply leaving the lights on where they are not needed. In addition, many people forget the lights on somewhere in the house, when going to bed or leaving the house. In these cases, it is easy to see where lights are on and to control them using the mobile app. In addition, a smart lighting control system supports home security by providing increased protection. For example, this means that while away on holiday, the lighting system could periodically switch on and off lighting in the house, as if someone were actually home. The light can be controlled through qToggle app, or using Google Home assistant and the voice command “turn on/off the light in ... room”. qToggle is compatible with Amazon Alexa as well. The devices used for controlling the lights are Sonoff Touch with one, two, or three channels.

### 3.3. Energy and Power Monitoring

**Figure 10:**
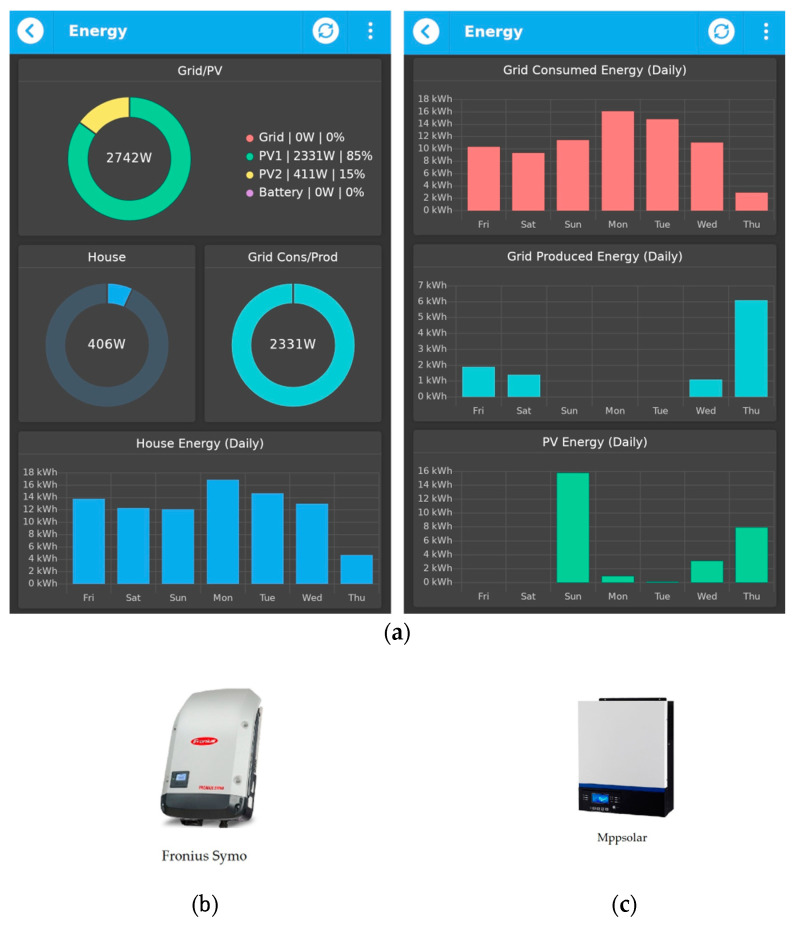
Monitoring the energy with qToggle (**a**) and the two invertors: Fronius Symo (**b**) and Mppsolar (**c**).

Nowadays, the whole world is looking for sustainable and energy efficient solutions to make our planet greener, so the use of renewable energy sources, such as solar energy to the maximum efficiency possible is the best solution. Photovoltaic panels convert the sun’s rays into electrical power and have become more affordable than ever. Combining the energy savings of solar systems with the smart technology, the benefit of renewable energy in a home is maximized. Home solutions can be fully automated using solar power. In addition to cutting energy bills and providing energy efficiency, solar power-based home solutions provide for the reduction of individual carbon footprint, give off zero emissions, and reduce overall environmental damage. For this case study, thirty-three photovoltaic (PV) panels have been installed, in two stages: the first sixteen panels (correspond to PV2 in Figure 10a, first panel, left), and then another seventeen panels (correspond to PV1 in Figure 10a, first panel, left). 

Solar installations require a dedicated solar inverter that converts solar power from the PV system into an alternating current. Inverters that are able to inject the excess of energy into the grid are called on-grid (or grid-tie) inverters and, in many countries, are subject to stricter rules than those that work off-grid. One of the most notable requirements for a grid-tie inverter is the anti-islanding protection: in case of a grid power outage, the inverter must immediately stop injecting energy, thus protecting electrical workers and upstream equipment. For this case study, the PV power inverters are from the following brand manufacturers: Fronius for PV1 (see Figure 10b) and MPPSolar for PV2 (see Figure 10c).

Solar energy can also be stored in batteries. When using batteries of a relatively large capacity, the energy accumulated over the day can be consumed during the night or during rainy days. Systems with smaller capacities may only be used as a backup, in case of grid outages, being able to supply the house with energy for a limited number of hours. An inverter that is capable of switching between grid, solar, and battery energy sources, depending on various configurable conditions, is called a hybrid inverter. MPPSolar is such a hybrid inverter.

**Figure 11:**
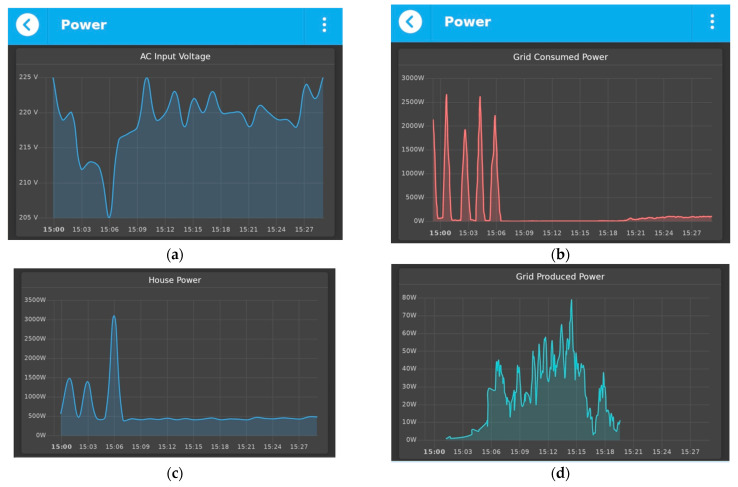
An example of monitoring the power with qToggle: AC Input Voltage (**a**); House Power (**b**); Gris Consumed Power (**c**); Grid Produced Power (**d**).

The aim for monitoring the power is to see how much energy the house is using and to become more aware of the energy use and, thus, of the money spent. An electricity monitor also helps identify any high energy appliance accidentally left switched on. Moreover, an important goal of power monitoring is the detection of abnormal conditions in voltage when the electrical network is undersized, and there are a lot of voltage variations. A smart power meter allows a continuous monitoring of all the important parameters when it comes to electricity: active, reactive, or apparent power, power factor, current, voltage, frequency, and total energy consumption. The device is based on ESP8266 and integrates a high current switch that can be used to remotely cut off energy supply, in case of an emergency. Figure 11 presents how the power is monitored using qToggle.

We consider that voltage monitoring is essential because the actual voltage supplied by the grid operator often varies from its nominal value, possibly causing faults to the electrical equipment. The chart (shown in Figure 11a) as well as its underlying historical data may serve as proof in case of appliance damage. The excess of solar produced power can be either used for household electrical necessities or it can be injected into the grid. Hence, we have the total house power (shown in Figure 11b), as well as the consumed and injected grid power (shown in Figure 11c,d).

### 3.4. Access Control and Security

**Figure 12:**
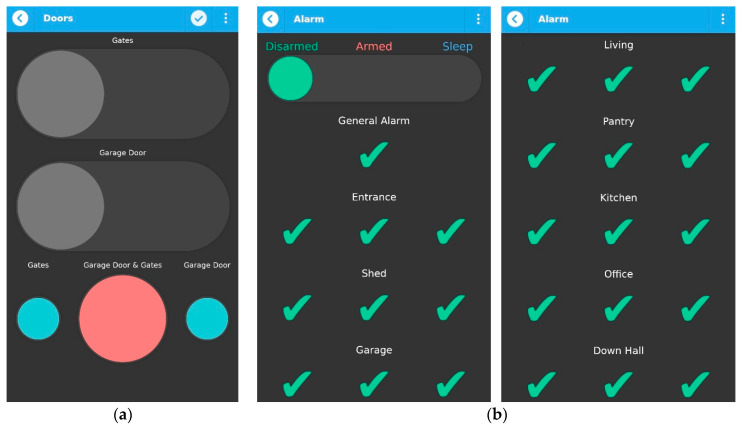
Access control (**a**) and security (**b**) with qToggle.

Access control involves controlling entrances, gates and doors, in this case study, the gates and the garage door, specifically. Various options can be chosen: to fully open or close only the gates, only the garage door, or both at the same time, or to keep half open one of them or both, as shown in Figure 12a. Access control can be done manually, using the app, and by vocal commands, using Google assistant on a smart watch. To control gate motors and the garage door, we used two Blitzwolf SS1smart relay boards that enable remote opening/closing. This allowed us to mimic the conventional gate remote control using our Wi-Fi-based system. 

The home security system consists of a master control panel, the keypad (when not using the qToggle app), motion sensors, and the siren. The qToggle app is provided with the options to arm and disarm the security system, as shown in Figure 12b. Arming and disarming can be performed by voice commands as well, using Google Assistant/smart watch. We have also implemented the Sleep option, which can be used during the night and arms only the ground floor of a house, for security reasons. If motion is detected downstairs during the night, the alarm will trigger. Arming and Disarming, using Sleep mode, can be done manually, using the qToggle app, but also using an extra light switch, placed upstairs (for more comfort). The alarm system used in this case study is a Paradox MG5050 alarm. A custom integration module has been developed using a Raspberry Pi board to be able to control the alarm unit remotely.

### 3.5. Controlling the Irrigations

**Figure 13:**
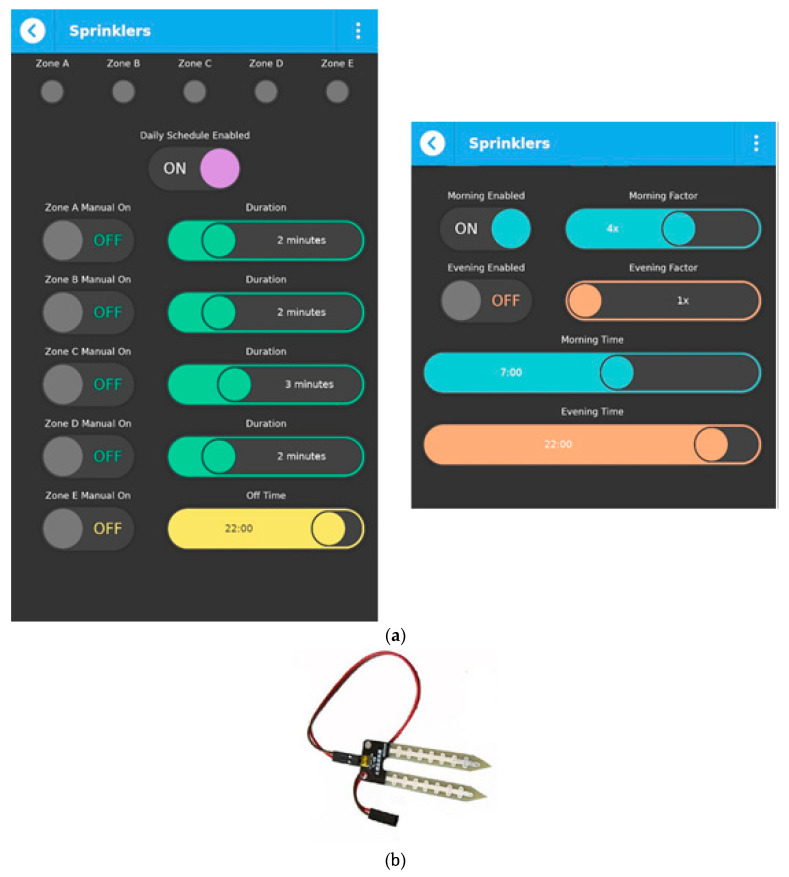
Controlling the irrigation system with qToggle (**a**) and the YL 69 sensor (**b**).

Automated irrigation systems help people control the water used in their gardens or fields and, thus, to avoid water waste, to save energy and time, and to minimize water bills. Using an automatic system based on valves instead of the classical manual irrigation also avoids human errors, for example forgetting to irrigate one day, not being able to do it, or forgetting to turn off the water after irrigation. The proposed irrigation system is based on Raspberry Pi and controls a number of pop-up sprinklers. The system inside the well contains: electric valves (Rain Bird DV/DVF valves), one standard 1.1 kW water pump, a Raspberry Pi board connected to the house LAN, and a pressure switch (Easy Press II model, with a maximum pressure of 10 bar). Figure 13a shows how the irrigation system can be controlled using the qToggle app. The user is able to select two modes: the manual one where he can start and stop irrigations whenever he wants and in which zone he wants and an automatic mode, with or without humidity sensors (YL69 sensors from SparkFun, in this case, shown in Figure 13b). 

In the automatic case, without sensors, the user is able to set schedules, by turning on the Enable schedule button. The adjustments can be made on the last four slides in the app: Morning factor, Evening factor, Morning time, and Evening time. The user is able to select the time in the morning and evening when the irrigation should start but also select the amount of water by adjusting the Morning/Evening factor. A time-controlled sprinklers system not only eliminates the chore of hand watering but also saves water, time, energy, and of course money. The sensors and actuators used for the presented case study, along with their main characteristics are shown in Table 3. Of course, in the arhitecture of qToggle, any kind of sensor or actuator can be used.

**Figure 14:**
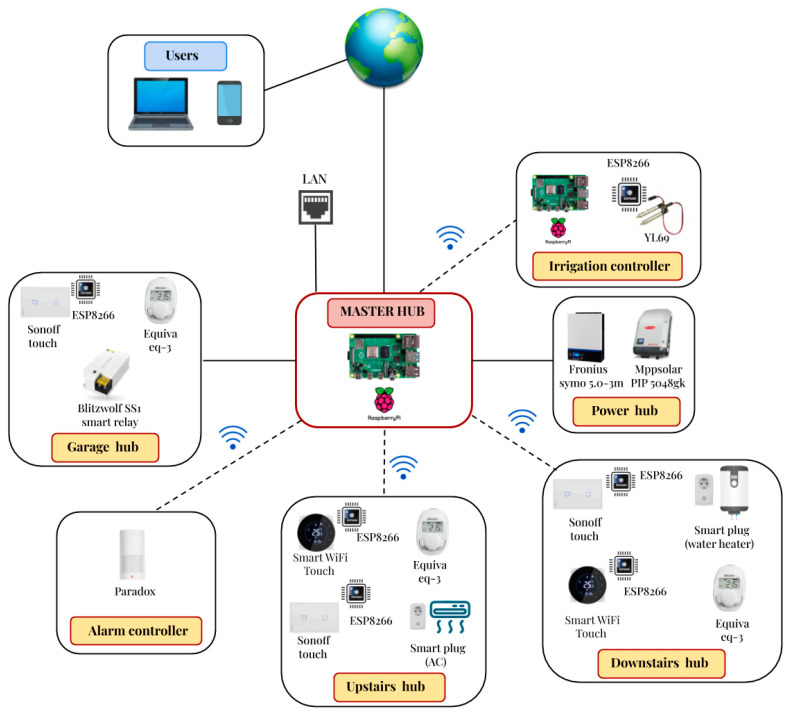
qToggle arhitecture for the presented case study.

The communication architecture for this case study is shown in Figure 14.

The qToggle architecture for this case study is composed of a Master hub connected to the house LAN and six hubs connected to the Master hub:

- Upstairs hub—controls lightning and HVAC systems upstairs
- Downstairs hub—controls lighting and temperatures downstairs
- Power hub—in charge with energy and power monitoring
- Garage hub—controls lights, temperature, and access
- Alarm controller—motion monitoring and alarm system control
- Irrigation controller—controls the irrigation system in the garden

The communication between the Master hub and the other hubs is done wirelessly except with the Garage hub and the Power hub, which is wired.

Interoperability and communication between devices is achieved by adopting a single, unitary, and extensible API across all devices that are part of a qToggle system.

For logging purposes, we use the Python logging module that captures logging from all modules and sends it to a log file, which is used for auditing and debugging purposes, if needed. System-level logs are gathered in the same manner into dedicated log files. Moreover, all log files are rotated on size and age conditions, so that we do not accidentally run out of storage due to excessive logging.

Events are actually inputs whose values change. These inputs are usually mapped to physical ports whose logic state may change, but they can also be virtual ports, which are obtained as combinations of other inputs, using Excel-like expressions.

## 4. Discussion and Future Work

In this project, we proposed a simple solution for home automation based on ESP8266 chips and Raspberry Pi boards. Both choices are cost effective, small, and easy to work with. Moreover, the proposed qToggle system uses a very basic core API, allowing for a more flexible network design. qToggle is aimed to be a complete smart home prototype, with a lot of functionalities—automation, control, monitoring, and security—and it is a system that could be continuously developed and improved. 

One contribution of this paper involves the reviewing of the recent (last 10 years) papers published in the literature, commercial solutions, and open source home automation systems (Table 1 and Table 2), so that the paper could be considered a survey. As compared to other papers in the literature, the proposed paper details the implementation of the solution (both hardware and software). Most smart home systems presented in the literature [4,5,6,7,8,9,10,11,12,13,14,15,16,17,18,19,20,21,22,23,24,25,26,27,28,29,30,31,32,33,34] have been made with fewer functionalities, using different technologies, controllers, type of communication, user interface, etc., and this is emphasized in Table 1. 

qToggle works with a selected list of devices, imposing a unitary API, firmware, and so on. We provide the open source firmware, meaning that no hacks and no 3rd party hubs or clouds are required; all devices speak the same language (API) and are controlled the same way. The supported devices are tested thoroughly, with a well-documented installation procedure. This does not mean that other devices cannot be added to qToggle: there are add-ons that provide bridges and adaptation layers to different peripherals, networks, and technologies. Regarding the number of devices, qToggle is highly scalable thanks to its master–slave architecture. One device can be at the same time a master to other slave devices and a slave for another master, at a higher place in the hierarchy. The core of qToggleServer, as well as the firmware that runs on ESP8266-based devices, are entirely asynchronous, meaning that a request can never block the functioning of the device. In turn, this allows for a relatively large number of incoming requests per second to each device, increasing the scalability of the system.

In this paper, we present a real case study (a real home) and all the features the proposed system (including the app) offers to make life easier and cheaper. The proposed solution can be implemented by any used using the code available on Github and can be used successfully in reality. This paper offers a description on how the system is implemented, how the app can be installed and configured, what functionalities are covered, and what devices can be used in order to have a smart home (see Figure 14).

qToggle’s strengths lie in its simplicity and flexibility. In addition, we intended to provide a low-cost home automation system. It is well known that the deployment and maintenance of a commercial home automation system come with a high cost. This cost is even higher if the number of devices that compose the system and used technologies grows. Usually, a basic package for automating a small house exceeds 1000 dollars. qToggle attempts to do a very good job at providing a curated list of supported devices. What motivates our approach is the fact that we control the firmware of each device (which is itself open source) and thus the entire user experience is a lot more integrated. Controlling the firmware also allows leveraging the full power of each device, customizing it per each user’s needs and providing critical security updates, while unifying the API. We take users’ privacy very seriously, and in qToggle, user data never leaves the premises (i.e., the home local network). Not being in control of the firmware of all devices, other platforms must rely on OEM or vendor-provided firmware, which often employs data communication via a 3rd party cloud service. Probably one of the biggest differences between qToggle and its competitors is that our target audience includes users without a technical background, who prefer intuitive, off-the-shelf solutions with minimal setup requirements and a smooth learning curve. qToggle provides a PWA (progressive web app). The advantages of a PWA include the fact that updates are immediate (not being served via App Store or Play Store) and there is one single code base shared among all platforms. Finally, maybe the most important thing is that we have provided a solution that can be used successfully in reality.

The future will bring an increase in terms of sensor products, as well as devices, thus automating every aspect of our home life. A feature that will be soon added to qToggle is monitoring the air humidity. Extreme humidity levels can cause mold and result in cost damage. A high humidity causes condensation and mold; a low humidity increases the risk of respiratory illnesses and allows viruses and germs to multiply. Thus, a humidity sensor will protect buildings and belongings by monitoring humidity levels, and it could be programmed to alert the customer in case the indoor humidity fluctuates to undesirable levels. 

Another future task will be the integration of video surveillance in qToggle. We have already developed a video surveillance OS for single board computers, so that a user can manage their video cameras very easily, in the browser. The system (called MotionEye) has become very popular in the open source world, with 50 releases on Github, and more than 650,000 downloads since 2014.

## Figures and Tables

**Table 1:** Features comparison for home automation system published in the last 10 years.

Home Automation System	Communication	Controller	User Interface	Applications
[4]	Bluetooth	PIC	mobile app	control indoor appliances
[5]	Bluetooth	Arduino	mobile app	control appliances indoor and outdoor, within short range
[6]	Bluetooth, GSM	PIC	mobile app	control appliances indoor and outdoor
[7]	ZigBee, Ethernet	Arduino MEGA	mobile app	control appliances indoor
[8]	X10, Serial, EIB, ZigBee, Bluetooth,	32-bit ARM microcontroller	Control panel (touch pad), desktop based	indoor automation solution
[9]	Wi-Fi, ZigBee	Raspberry PI, NodeMCU		controlling humidity, temperature, luminosity, movement, and current
[10]	ZigBee	Laptop/PC server	mobile app	control of indoor appliances but not actually implemented
[11]	ZigBee, Wi-Fi	Linux board	GUI interface	control HVAC appliances
[12]	ZigBee, Wi-Fi, Ethernet	Raspberry PI	web-based, mobile app	remote control of appliances (IP cams, smart plugs)
[13]	Wi-Fi	TI-CC3200 MCU	mobile app	control indoor appliances, monitor the soil moisture
[14]	Wi-Fi	NodeMCU	web-based	control indoor appliances
[15]	Bluetooth, Wi-Fi	Raspberry PI	mobile app	control indoor appliances
[16]	Wi-Fi	Arduino mega	web-based, mobile app	control of indoor appliances
[17]	Wi-Fi	PC server	web-based, mobile app	security, energy management
[18]	Wi-Fi, IR	PC server	mobile app	control of indoor appliances
[19]	Wi-Fi	Arduino	mobile app	control indoor appliances, video surveillance
[20]	Bluetooth	Arduino	mobile app	control indoor appliances, energy management
[21]	Wi-Fi	Arduino, ESP8266	mobile app	control indoor appliances
[22]	Bluetooth, Wi-Fi	Arduino mega	web-based, mobile app	indoor and outdoor control, monitoring, energy management, safety, security
[23]	Ethernet	Arduino mega	web-based	control of indoor appliances
[25]	Ethernet	Raspberry PI	web-based	control home appliances, surveillance
[26]	ZigBee, Z-wave, Wi-Fi	Raspberry PI	unspecified	light automation and physical intrusion detection
[27]	Wi-Fi	NodeMCU	web-based, mobile app	control indoor appliances (luminosity sensor, LED, buzzer)
[28]	Wi-Fi	ESP8266	unspecified	testing modules in a smart home system, related to indoor appliances control, surveillance, energy management
[29]	Wi-Fi	Arduino, ESP8266	mobile app	control of switches
[30]	Wi-Fi	Node MCU	web-based, mobile app	control of appliances indoor and outdoor, safety, security, energy management, monitoring
[31]	Ethernet	Galileo board	web-based, mobile app	indoor and outdoor control, energy management, security
[32]	GSM, Wi-Fi	PC server	web-based	safety, monitoring (gas, temperature, fire sensors)
[33]	GSM	8051 MCU	web-based	indoor and outdoor control
[34]	GSM	Arduino	web-based	control of indoor appliances, safety, energy management
[35]	ZigBee, Wi-Fi, GSM/GPR	PC	LabVIEW PDA Module	remote monitoring and control system for intelligent buildings
[36]	ZigBee	PC	web app	power outlet control
**qToggle**	**Wi-Fi**	**Raspberry PI, ESP 8266**	**web-based, mobile app**	**multiple home automations** **indoor and outdoor, irrigations, security, monitoring, power and energy management (including solar energy), Google assistant compatible**

**Table 2:** Comparison of the most relevant open-source home automation platforms.

System	Development Language	API	Other Features
OpenHAB	Java	Representational state transfer (REST)	web interface, many protocols, many plugins, MQTT, EPL v1 license, extensive documentation
HomeAssistant	Python	REST/Python/Websocket APIs	web interface, many protocols, many plugins, MQTT, Apache 2.0 license, extensive documentation
Domoticz	C++	JSON based	web interface, many protocols, many plugins, MQTT, GPL v3 license, extensive documentation
Calaos	C++	JSON based	web interface, a few protocols, under development plugins, MQTT, GPL v3 license, extensive documentation (in French)
Jeedom	PHP	JSON RPC and HTTP based	web interface, many protocols, many plugins, MQTT, GPL v2 license, extensive documentation (in French)
Fhem	Perl	ASCCII commands	web interface, many protocols, many plugins, MQTT, GPL v2 license, extensive documentation (in German)
**qToogle**	**Python**	**JSON based REST**	**web interface, many protocols, a few plugins (undergoing continuous development), Hypertext Transfer Protocol (HTTP) based messaging, Apache 2.0 license, extensive documentation (in English on Github)**

**Table 3:** Sensors and actuators used by qToggle for this case study.

Sensor/Actuator	Name	Price	Measurement Range	Consumption
Temperature	ME81H	$25–30	5–60 °C	450 mA
Soil moisture	YL69	$5	0–100%	35 mA
Motion	Paradox PIR sensor	$35–40	logic	ultralow power
Power meter	ZMAi-90	$30–35	90–250 V, 0–60 A, 0–15 kW	1 W
Simple relay board	Blitzwolf SS1	$8		0.5 W
4-relay module	SainSmart	$3–5		5–65 mA
Touch sensor	Sonoff Touch	$15–20	logic	0.5 W

## Abbreviations

The following abbreviations are used in this manuscript:

API	Application Programming Interface
HVAC	Heating, Ventilation and Air Conditioning
HTTP	Hypertext Transfer Protocol
IoT	Internet of Things
JSON	JavaScript Object Notation
MQTT	Message Queuing Telemetry Transport
PWA	Progressive Web App
REST	Representational state transfer
TCP-IP	Transmission Control Protocol-Internet Protocol
